# Loss of alpha-smooth muscle actin expression associated with chronic intestinal pseudo-obstruction in a young Miniature Bull Terrier

**DOI:** 10.1186/s13028-018-0379-3

**Published:** 2018-04-24

**Authors:** Gian Enrico Magi, Francesca Mariotti, Sara Berardi, Andrea Piccinini, Cecilia Vullo, Angela Palumbo Piccionello, Giacomo Rossi

**Affiliations:** School of Biosciences and Veterinary Medicine, University of Camerino, Via Circonvallazione 93/95, 62024 Matelica, MC Italy

**Keywords:** Chronic intestinal pseudo-obstruction, Dog, Intestinal fibrosis, Myopathy

## Abstract

**Background:**

Chronic intestinal pseudo-obstruction (CIPO) is a rare clinical syndrome in veterinary medicine characterized by severe intestinal dysmotility without evidence of mechanical occlusion of the intestinal lumen. The exact pathogenesis of CIPO is unknown.

**Case presentation:**

A 1-year-old male Miniature Bull Terrier dog was presented with a history of chronic weight loss, regurgitation, lethargy, vomiting and diarrhea. The dog was submitted for exploratory laparotomy. A full thickness intestinal biopsy was taken and a CIPO was suspected. The clinical condition deteriorated and the dog was euthanized. At gross examination the small intestine was severely dilated. Histologically severe fibrosis of the submucosa and severe atrophy of the tunica muscularis were present in small intestine and colon. Immunohistochemical examination with a panel of antibodies for gastro-intestinal neuromuscular disease-associated antigens revealed a severely reduced expression of alpha-smooth muscle actin in the tunica muscularis.

**Conclusions:**

This case report describes the gross, histological and immunohistochemical findings of CIPO affecting a 1-year-old Miniature Bull Terrier; on the basis of these findings a myopathic form of CIPO is hypothesized in this case.

## Background

Chronic intestinal pseudo-obstruction (CIPO) is a rare clinical syndrome in veterinary medicine characterized by severe intestinal dysmotility without evidence of mechanical occlusion of the intestinal lumen. In the veterinary literature, few cases of CIPO affecting dogs, horses and cats have been reported [1–10]. In human medicine, CIPO can be caused by different gastrointestinal neuromuscular diseases (GINMDs) including primary visceral neuropathies, interstitial cell diseases and myopathies, where different neurointerstitial-muscular components are involved [11, 12].

In dogs, most cases have been related to an idiopathic fibrosing enteropathy or fibrosing gastrointestinal leiomyositis, or less frequently to dysautonomia [4, 5, 7–10, 13]. Due to the rarity of this condition and appropriate studies, the pathogenic mechanisms are still poorly understood. This case report describes the gross, histological and immunohistochemical findings in a young Mini Bull Terrier affected by CIPO.

## Case presentation

A 1-year-old male Miniature Bull Terrier dog was presented with a 2-month history of chronic weight loss, regurgitation, lethargy, vomiting and diarrhoea. The dog received prokinetic treatment, antiemetics, antimicrobial agents, and intravenous fluid therapy, without significant clinical improvement. Abdominal radiographs revealed marked small intestinal dilation. On exploratory laparotomy, the small intestine was not obstructed but appeared markedly distended and a full-thickness biopsy of the jejunum was taken. Histological examination revealed severe atrophy of the tunica muscularis and fibrosis of the tunica submucosa and a diagnosis of CIPO or neuromuscular disorder was suspected. Following the histopathological examination, the dog was unsuccessfully treated with glucocorticoids. Due to the persistent clinical signs of dysmotility, the dog’s clinical condition severely deteriorated thus euthanasia was elected.

**Fig. 1:**
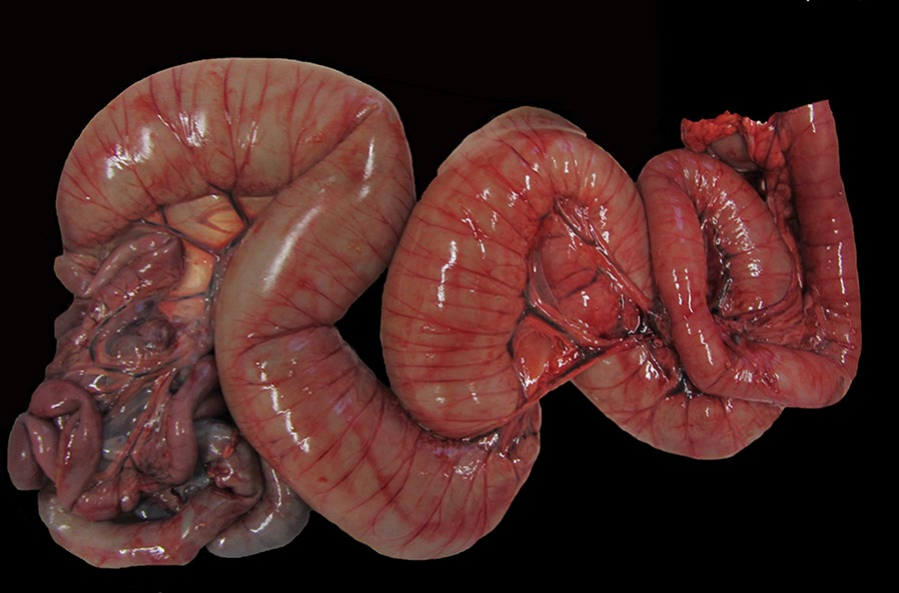
Small intestine with diffuse severe dilation

At necropsy the dog appeared dehydrated, in poor nutritional condition and with minimal body fat amounts. The small intestine was severely dilated and filled by a variable amount of grey-greenish fluid (Fig. 1). The esophagus, stomach and the large intestine were also dilated but to a lesser extent. The small intestinal wall was diffusely thinned and atonic. Tissues samples, including specimens of the thoracic and abdominal organs, the skin and skeletal muscle were taken for histopathology as were parts of the oesophagus, the stomach, the duodenum, the jejunum, the ileum, the cecum, the colon and the rectum. The samples were fixed in 4% neutral-buffered formaldehyde for 48 h. After fixation, the specimens were processed by an automatic histoprocessor and embedded in paraffin. Serial sections (3 µm) were stained with hematoxylin and eosin (HE), periodic acid-Schiff (PAS) technique and Masson’s trichrome method. For immunohistochemistry, sections of stomach, duodenum, jejunum, ileum, cecum, colon and rectum tract were mounted on Superfrost^®^Plus slides and an avidin–biotin–peroxidase-complex (ABC) technique with diaminobenzidine as the chromogen was performed. A panel of antibodies to the following antigens was used: alpha-smooth muscle actin (α-SMA) (smooth muscle cells, myofibroblast; monoclonal, clone 1A4, Sigma-Aldrich, St. Louis, USA), CD3 (T-lymphocytes; monoclonal, clone CD3-12 Bio-Rad, UK), CD79a (B-lymphocytes; monoclonal, clone HM57, Bio-Rad, UK), CD117 (interstitial cells of Cajal, polyclonal, Santa Cruz Biotechnology, USA), glial fibrillary acid protein (GFAP) (enteric glial cells; polyclonal, Dako, Glostrup, Denmark), neurofilaments (neurons; monoclonal, clone2F11, Dako), neuron specific enolase (NSE) (neuronal and neuroendocrine cells; polyclonal, Calbiochem, USA), S-100 (enteric glial cells; polyclonal, Dako), and synaptophysin (neuronal and neuroendocrine cells; monoclonal, Dako). Appropriate negative and positive controls including gastrointestinal tissue sections of an healthy dog were used for the immunohistochemical analyses. The specificity of the different antibodies was evaluated considering the information available in the datasheet for each antibody. Moreover, the specificity and cross-reactivity of the antibodies were evaluated based on published studies [14–19].

**Fig. 2:**
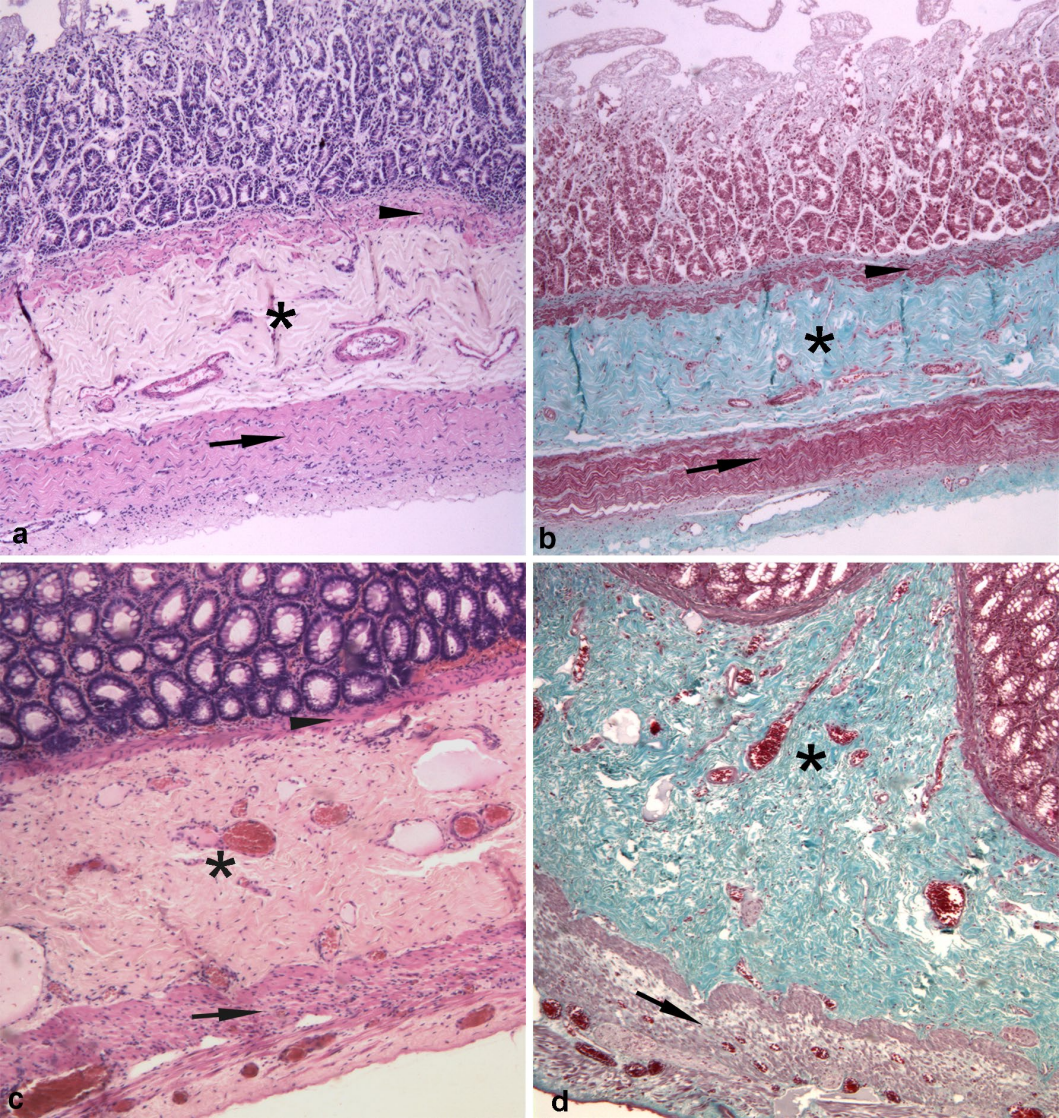
**a**, **b** Jejunum. Severe fibrosis of the tunica submucosa (asterisk), hypertrophy of the lamina muscularis mucosae (arrowhead) and severe atrophy of the tunica muscularis (arrow). **c**, **d** Colon. Severe fibrosis of tunica submucosa (asterisk), hypertrophy of the lamina muscularis mucosae (arrowhead) and severe atrophy of tunica muscularis (arrow). (**a** and **c**: HE; **b** and **d**: Masson’s trichrome, obj. ×4)

**Fig. 3:**
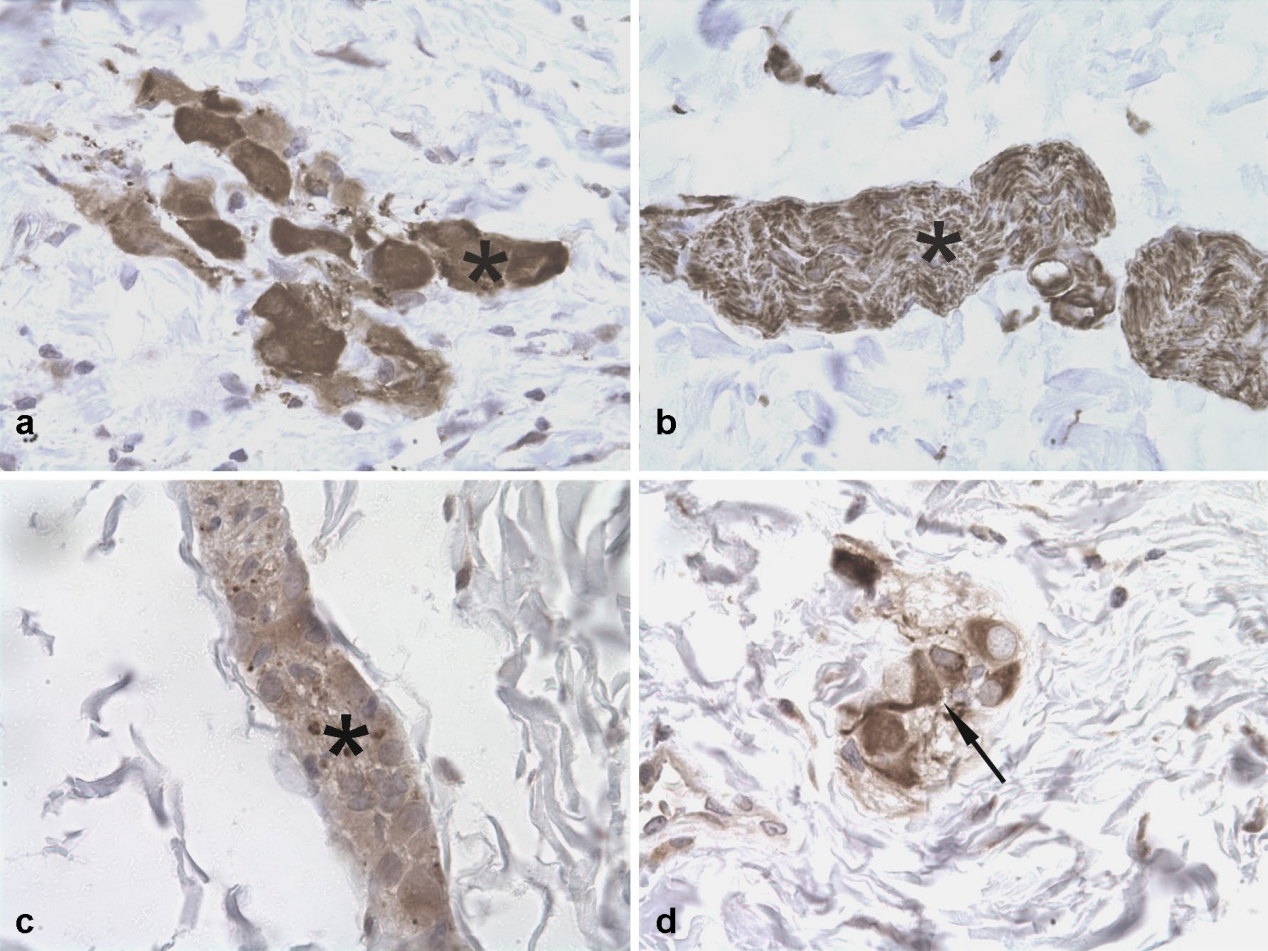
Immunohistochemical findings in the jejunum. **a** Submucosal nerve plexuses with neurons positive for NSE (asterisk). **b** Submucosal nerve plexuses immunopositive for neurofilaments (asterisk). **c** Submucosal nerve plexuses immunopositive for synaptophysin (asterisk). **d** Submucosa. Interstitial cells of Cajal immunostained for CD117 (arrow). *IHC* counterstained with hematoxylin (obj. ×4)

**Fig. 4:**
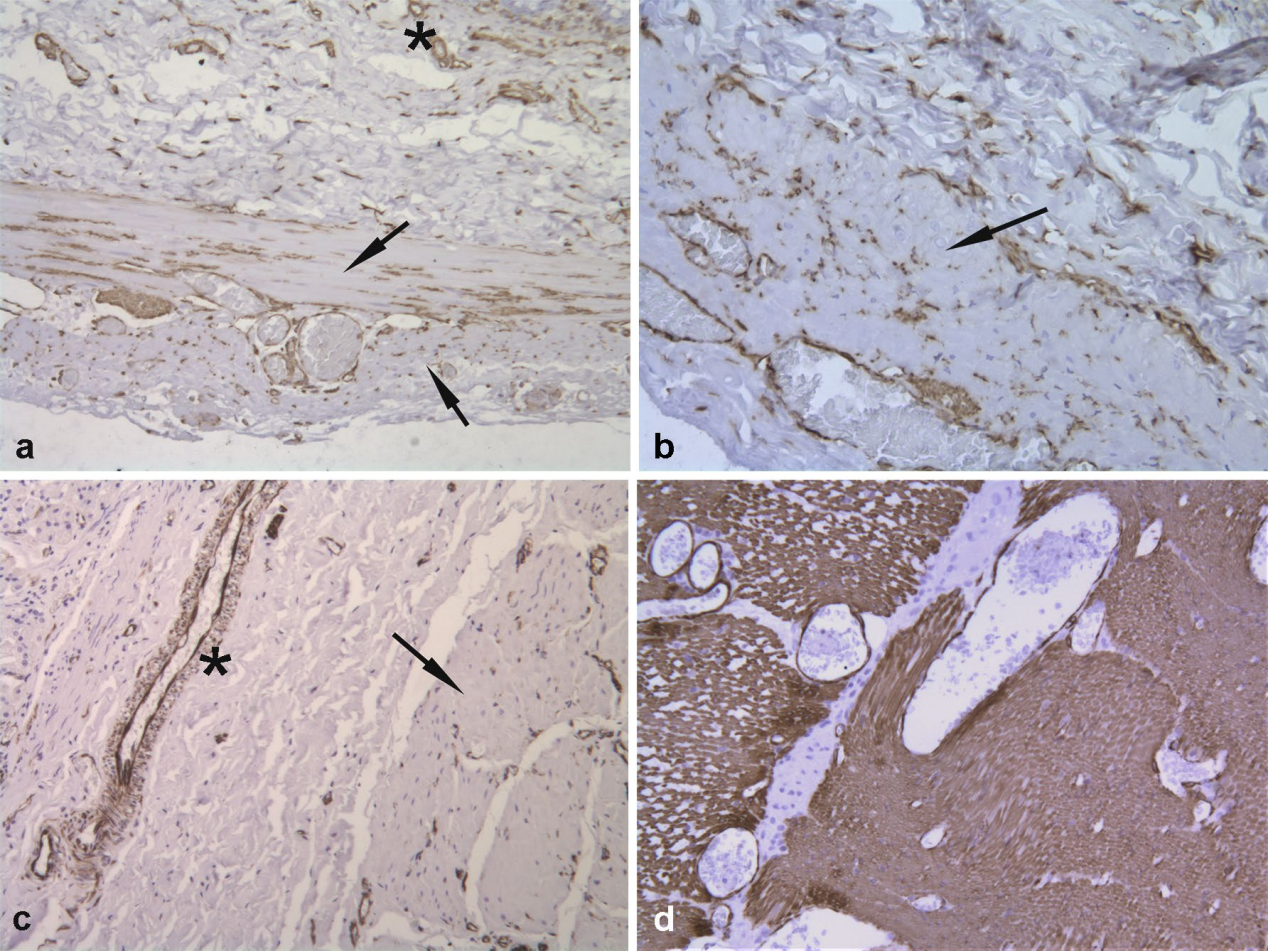
Immunohistochemical findings for α-SMA. **a** Jejunum. Diffuse low expression within the inner circular layer and the longitudinal layer of tunica muscularis. (arrow) Small vessels appear positive for α-SMA (asterisk). **b** Colon. Diffuse reduced expression within the tunica muscularis (arrow). **c** Stomach. Diffuse loss of expression within the tunica muscularis (arrow). Wall of a medium vessel positive for α-SMA (asterisk). **d** Normal canine duodenum. Diffuse intense α-SMA expression of the tunica muscularis. **a**–**d** *IHC* counterstained with haematoxylin (obj. ×10)

Histological findings for duodenum, jejunum, ileum, cecum, colon and rectum consisted of severe diffuse atrophy of the tunica muscularis and severe locally-extensive to diffuse fibrosis of tunica submucosa as demonstrated by Masson’s trichrome stain (Fig. 2a–d). Similar but less severe fibrosis was present within the submucosa of the stomach. The lamina muscularis mucosae appeared moderately hypertrophic (Fig. 2a–d). Additionally, the intestinal mucosa of duodenum and ileum appeared multifocally eroded and within the tunica submucosa and the tunica muscularis, multifocal moderate to severe lymphangiectasia was observed. The myenteric and submucosal nerve plexuses of all segments of the gastrointestinal tract had intact neurons confirmed by immunohistochemistry for NSE, neurofilaments and synaptophysin (Fig. 3a–c). Few lymphocytes as well as cells staining positive for GFAP were observed within interstitium or within myenteric plexuses respectively. Also interstitial cells of Cajal were preserved and were strongly stained for CD117 (Fig. 3d). However, α-SMA immunoreactivity was diffusely and markedly reduced in the muscular layers of all gastrointestinal segments examined with foci of complete loss compared to the control (Fig. 4a–d).

## Discussion and conclusions

In veterinary medicine, most of the reported cases of canine CIPO were histologically characterized by fibrosis and muscular atrophy of the gastro-intestinal tract along with variable inflammation centred on the tunica muscularis (leiomyositis) [4, 5, 7, 8]. In the present case, there was segmental fibrosis of the submucosa and severe atrophy of tunica muscularis but the inflammatory component was minimal, similarly to other canine CIPO cases [1, 10, 20]. This finding was also observed in the initial intestinal biopsy when the corticosteroid therapy had not yet been initiated. Hypertrophy of the lamina muscularis mucosae was present as also reported by others [1, 10, 20].

In human medicine, immunohistochemistry is used in order to discriminate between different forms of CIPO: myopathic, neuropathic and those caused by abnormalities of the interstitial cells of Cajal, by means of a panel of antibodies able to identify which neurointerstitial-muscular component is involved [11, 12, 21]. In numerous human GINMD cases, loss of α-SMA immunoreactivity was the only finding and has been associated with CIPO [21–23]. In human medicine, the consensus statement created by an international working group defined diagnostic algorithms and techniques for evaluating GINMD and consider loss of α-SMA expression as a marker for a myopathic form of CIPO [11, 12, 21]. Whether an α-SMA abnormality is the cause of altered motility is unknown. Recently, a case of CIPO associated with deficient expression of α-SMA in the muscular layer and loss of myofibrils has been described in a Bengal cat and a leiomyopathy was hypothesized [24]. In our case, the loss α-SMA immunoreactivity was diffuse affecting both the inner circular layer and longitudinal layer of the tunica muscularis and the lamina muscularis mucosae in contrast to the human cases and the feline case where only the circular layer appeared involved.

Neuropathy was not considered in our case due to the lack of enteric nervous plexus inflammation and the absence of neuronal degeneration. Additionally, the dog did not exhibit clinical signs consistent with dysautonomia. The clinical, histopathological and immunohistochemical findings of this rare case diagnosed with CIPO is consistent with enteric myopathy and intestinal fibrosis and could be referred to a myopathic form of GINMDs. In another CIPO case affecting a Pug, there was also a marked segmental loss of α-SMA expression in the intestinal muscular layer [20]. These findings suggest a possible crucial role of α-SMA in the pathogenesis of this still poorly understood condition.

In conclusion, the present case report describes the first CIPO case described in a Miniature Bull Terrier and highlights the importance of immunohistochemical examination in order to characterize the type of GINMDS and add new information about the underlying pathogenic mechanisms. Further studies at the genetic level are necessary to explain if loss of α-SMA expression is associated with a specific genetic disorder.

## Abbreviations

α-SMA: alpha-smooth muscle actin
ABC: avidin–biotin–peroxidase-complex
CIPO: chronic intestinal pseudo-obstruction
GFAP: glial fibrillary acid protein
HE: hematoxylin and eosin
GINMDs: gastrointestinal neuromuscular diseases
NSE: neuron specific enolase
PAS: periodic acid-Schiff
